# Supplementary material for: Variation in the fruit development gene POINTED TIP regulates protuberance of tomato fruit tip
Source: Nat Commun. 2022 Oct 8;13:5940. doi: 10.1038/s41467-022-33648-4 (PMC9547884; doi:10.1038/s41467-022-33648-4)
Supplement: Supplementary file 1 — Supplementary Information [file 41467_2022_33648_MOESM1_ESM.pdf]

Variation in the fruit development gene *POINTED TIP* regulates protuberance of tomato fruit tip

Song *et al.*

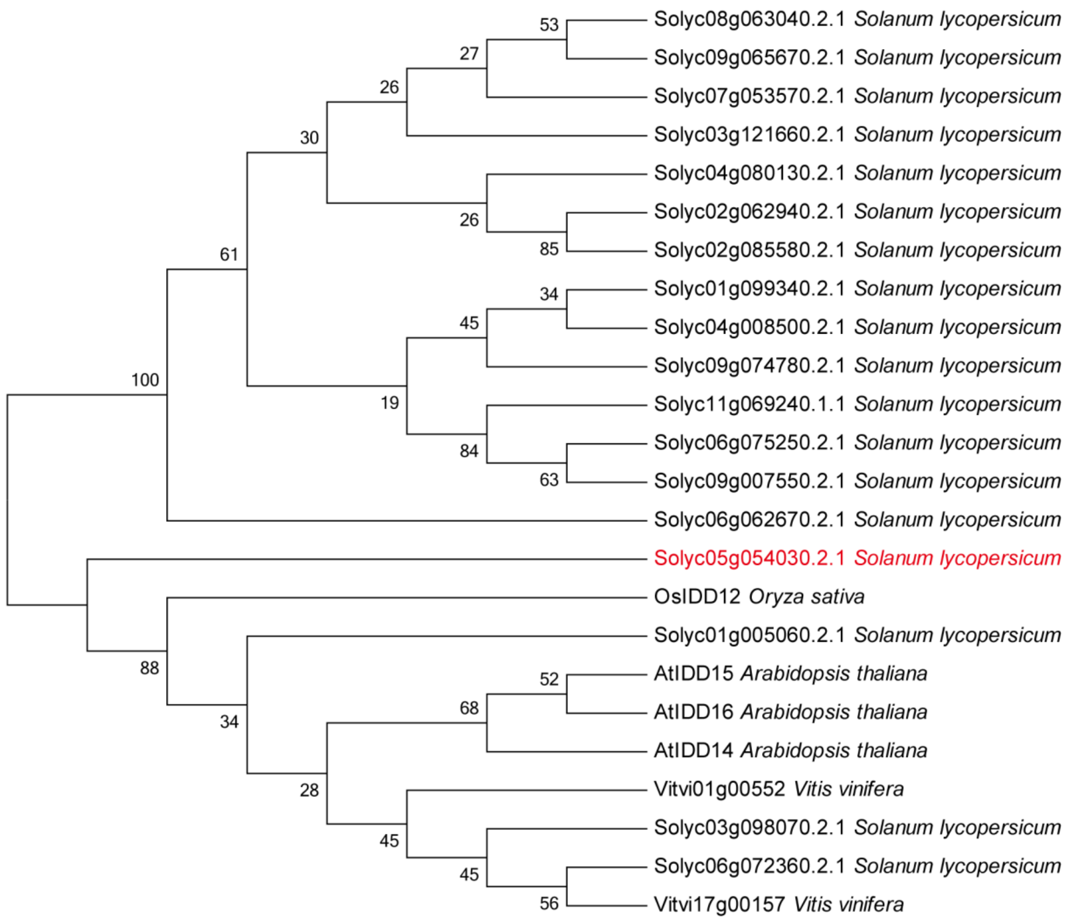

Supplementary Fig. 1. Phylogenetic tree analysis of PT.

Phylogenetic relationships of PT proteins from *Solanum lycopersicum*, *Arabidopsis thaliana*, *Oryza sativa*, and *Vitis vinifera*. The full-length amino acid sequences of PT paralogous and orthologous were downloaded from EnsemblPlants and aligned using Clustal W2. The phylogenetic tree was constructed using the neighbor-joining algorithm in MEGA 7. The red ID indicates PT.

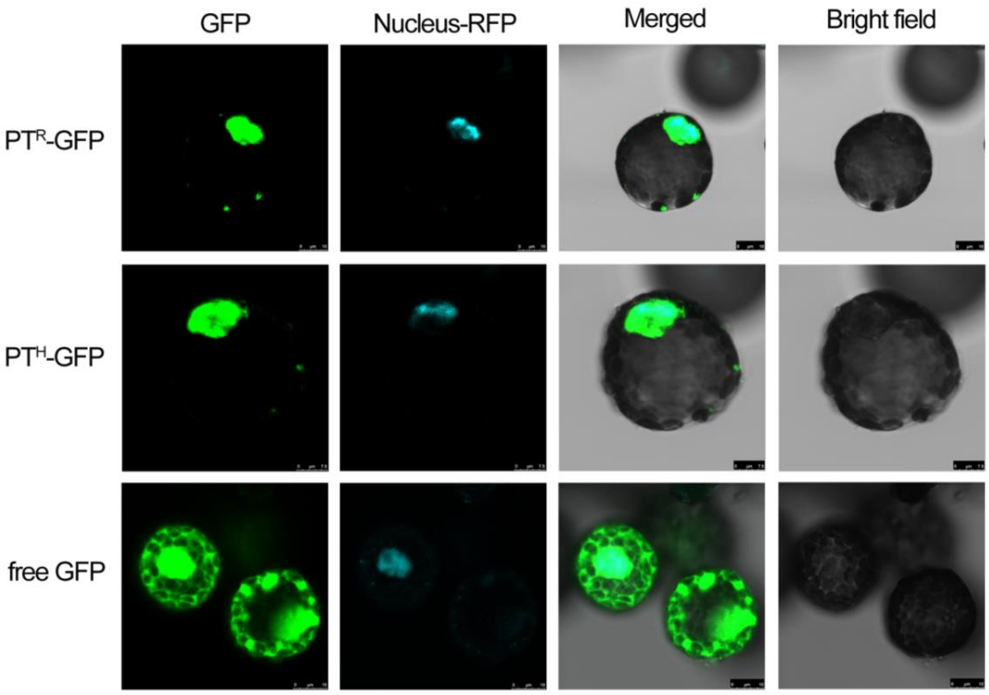

Supplementary Fig. 2. Subcellular localization of PT^R and PT^H proteins.
Tobacco protoplasts were co-transformed with plasmids that express either PT^R-GFP or PT^H-GFP and a nuclear marker Ghd7-CFP. Free GFP served as a control. Green and cyan signals indicate fluorescence from GFP and the nuclear marker, respectively. Three independent experiments were performed.

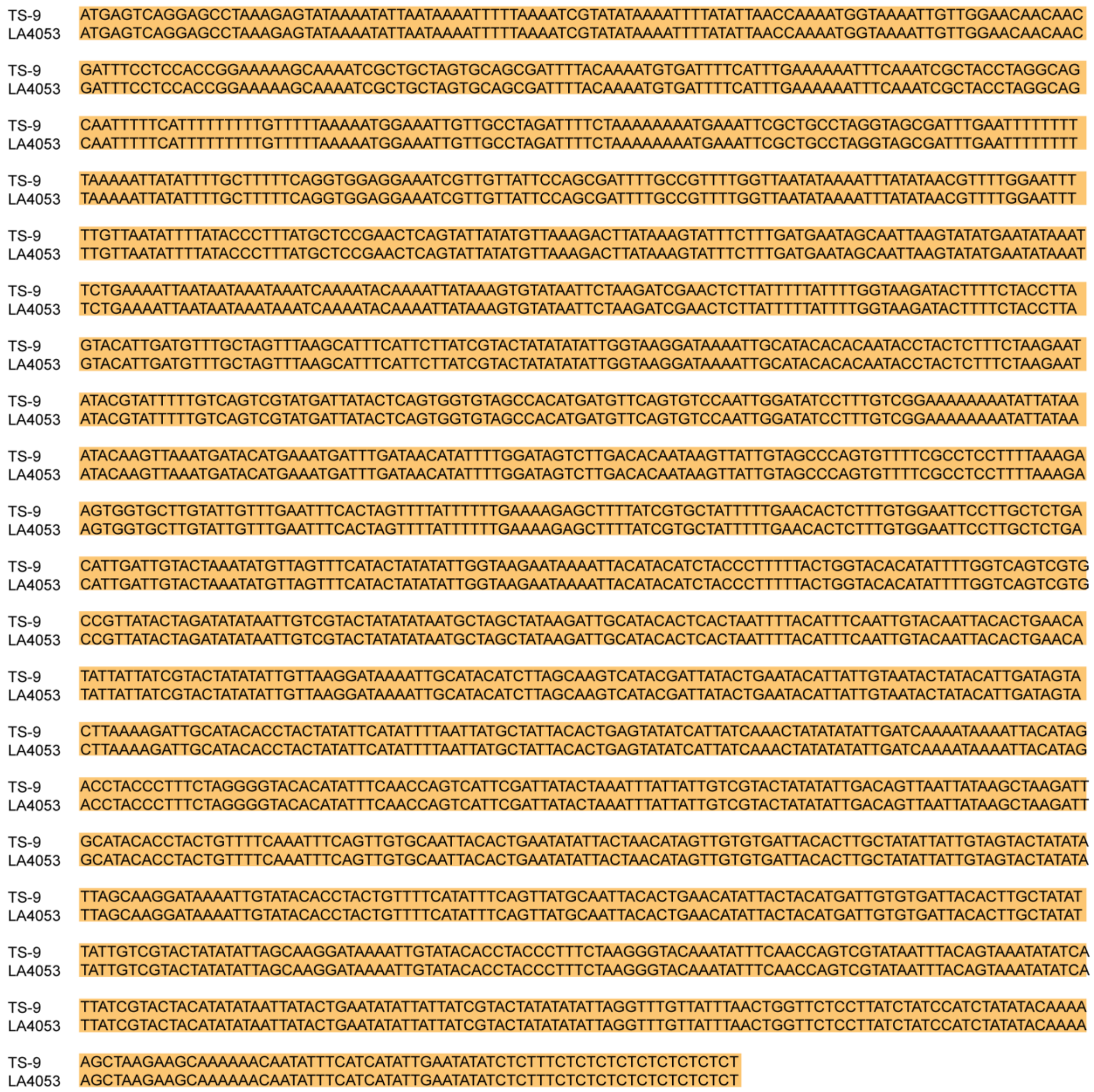

Supplementary Fig. 3. Alignment of the *PT* promoter sequences.

TS-9 is an accession that produces non-pointed tip fruit. LA4053 is an accession that produces fruit with pointed tips.

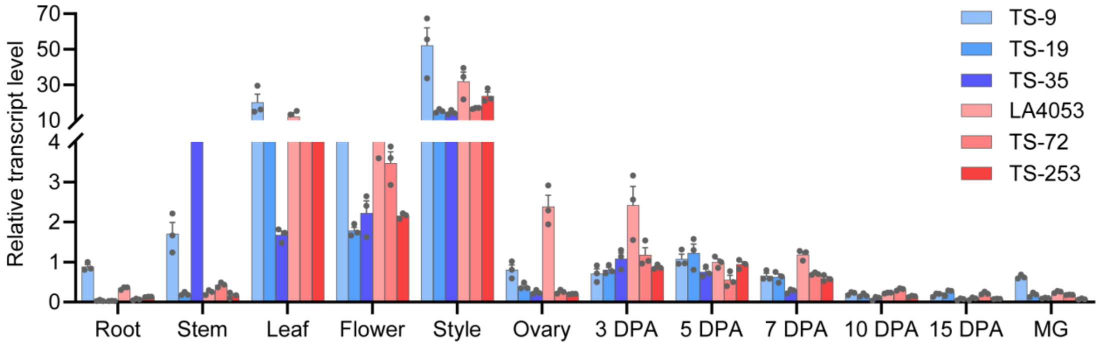

Supplementary Fig. 4. Expression patterns of *PT*.

Relative transcript levels of *PT* in different tissues from pointed tip accessions and non-pointed tip accessions. TS-9, TS-19, and TS-35 produced fruit with non-pointed tips. LA4053, TS-72, and TS-253 developed pointed tip fruit. DPA, day post anthesis; IG, immature green. Error bars indicate mean ± SE. *n*=three biological replicates. Source data are provided as a Source Data file.

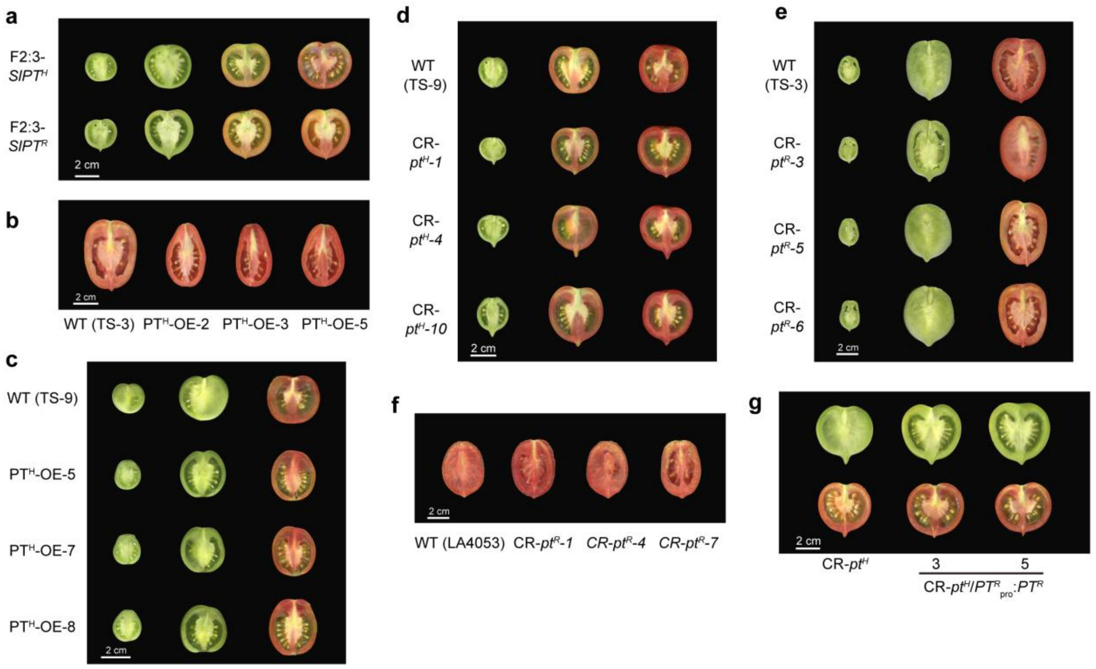

Supplementary Fig. 5. Longitudinal sections from fruit produced by different transgenic lines and pertinent wild-type plants.

a Fruit from plants harboring the *PT*^{R} and *PT*^{H} alleles from the F2:3 population. **b**, **c** Fruit from *PT*^{H}-overexpressing lines (**b**: *PT*^{H}-OE-2, *PT*^{H}-OE-3, and *PT*^{H}-OE-5) and its pertinent wild type control (TS-3 *PT*^{R} +/+), (**c**: *PT*^{H}-OE-5, *PT*^{H}-OE-7, and *PT*^{H}-OE-8) and the pertinent wild-type line (TS-9 *PT*^{H} +/+). **d**-**f** Fruit from CR-*pt*^{H} mutants (CR-*pt*^{H}-1, CR-*pt*^{H}-4, and CR-*pt*^{H}-10) and pertinent wild type control (TS-9) that produces non-pointed tip fruit (**d**), CR-*pt*^{R} mutants (CR-*pt*^{R}-3, CR-*pt*^{R}-5, and CR-*pt*^{R}-6) and its pertinent wild type control (TS-3) that produces fruit with pointed tips (**e**), CR-*pt*^{R} mutants (CR-*pt*^{R}-1, CR-*pt*^{R}-4, and CR-*pt*^{R}-7) and the pertinent wild type control (LA4053) that produces fruit with a pointed tip (**f**). **g** Fruit from CR-*pt*^{H}/*PT*$^{R}_{pro}$:*PT*^{R} lines (CR-*pt*^{H}/*PT*$^{R}_{pro}$:*PT*^{R}-3 and CR-*pt*^{H}/*PT*$^{R}_{pro}$:*PT*^{R}-5) and its pertinent control (CR-*pt*^{H}).

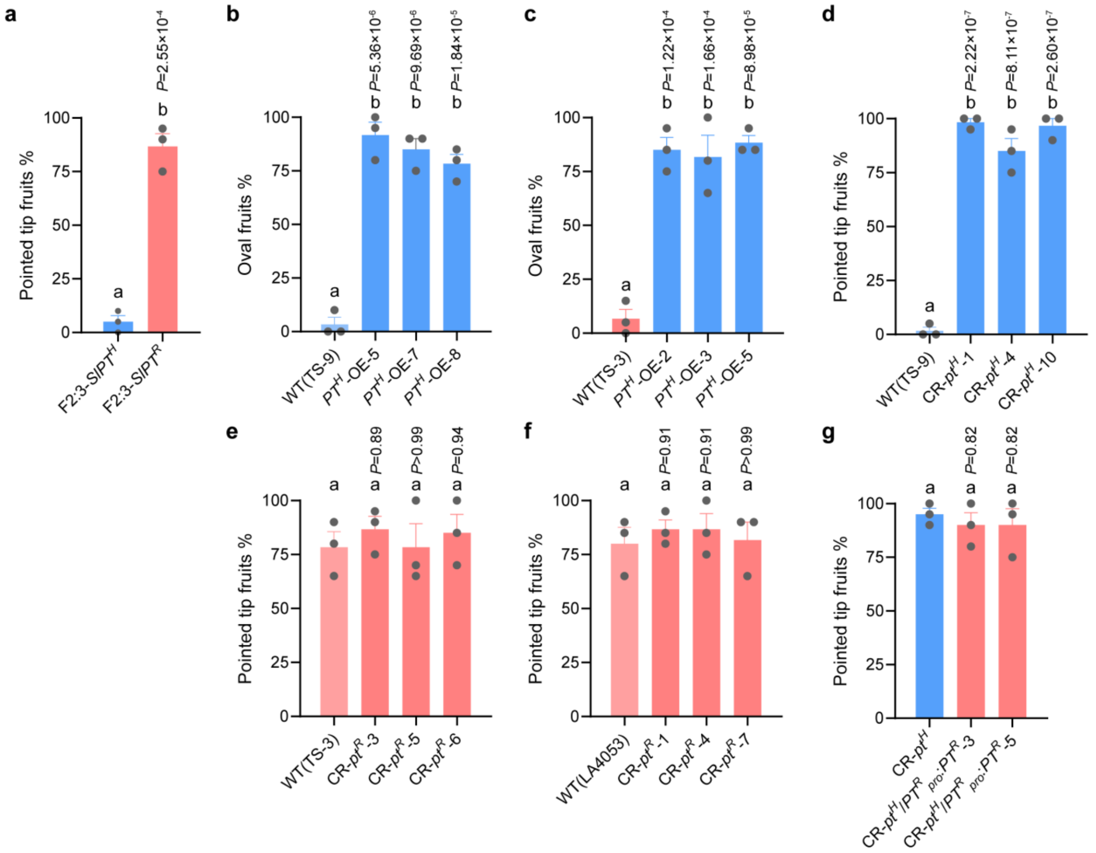

Supplementary Fig. 6. Percentage of different fruit morphology produced by different transgenic lines and their pertinent wild-type plants.

a Percentage of pointed tip fruits from plants harboring PT^R and PT^H alleles from the F2:3 population. **b**, **c** Percentage of oval fruits from PT^H-overexpressing lines (PT^H-OE-5, PT^H-OE-7, and PT^H-OE-8) and pertinent wild-type (TS-9, PT^H allele, **b**), PT^H-overexpressing lines (PT^H-OE-2, PT^H-OE-3, and PT^H-OE-5) and pertinent wild-type (TS-3, PT^R allele, **c**). **d-f** Percentage of pointed tip fruits from CR-pt^H mutants (CR-pt^H-1, CR-pt^H-4, and CR-pt^H-10) and pertinent wild type control (TS-9, **d**), CR-pt^R mutants (CR-pt^R-3, CR-pt^R-5, and CR-pt^R-6) and its pertinent wild type control (TS-3, **e**), CR-pt^R mutants (CR-pt^R-1, CR-pt^R-4, and CR-pt^R-7) and the pertinent wild type control (LA4053, **f**). **g** Percentage of pointed tip fruits from CR-pt^H/PT^R_{pro}:PT^R lines (CR-pt^H/PT^R_{pro}:PT^R-3 and CR-pt^H/PT^R_{pro}:PT^R-5) and its pertinent control (CR-pt^H). 20 fruits from each replicate were harvested and recorded for the morphologies (pointed tip, non-pointed tip or oval). Error bars indicate mean ± SE. *n*=three biological replicates. Statistically significant differences were determined using a two-tailed *t* test (**a**) and one-way ANOVA with Tukey's post-hoc test (**b-g**). Different letters indicate statistically significant differences ($P < 0.05$). Source data are provided as a Source Data file.

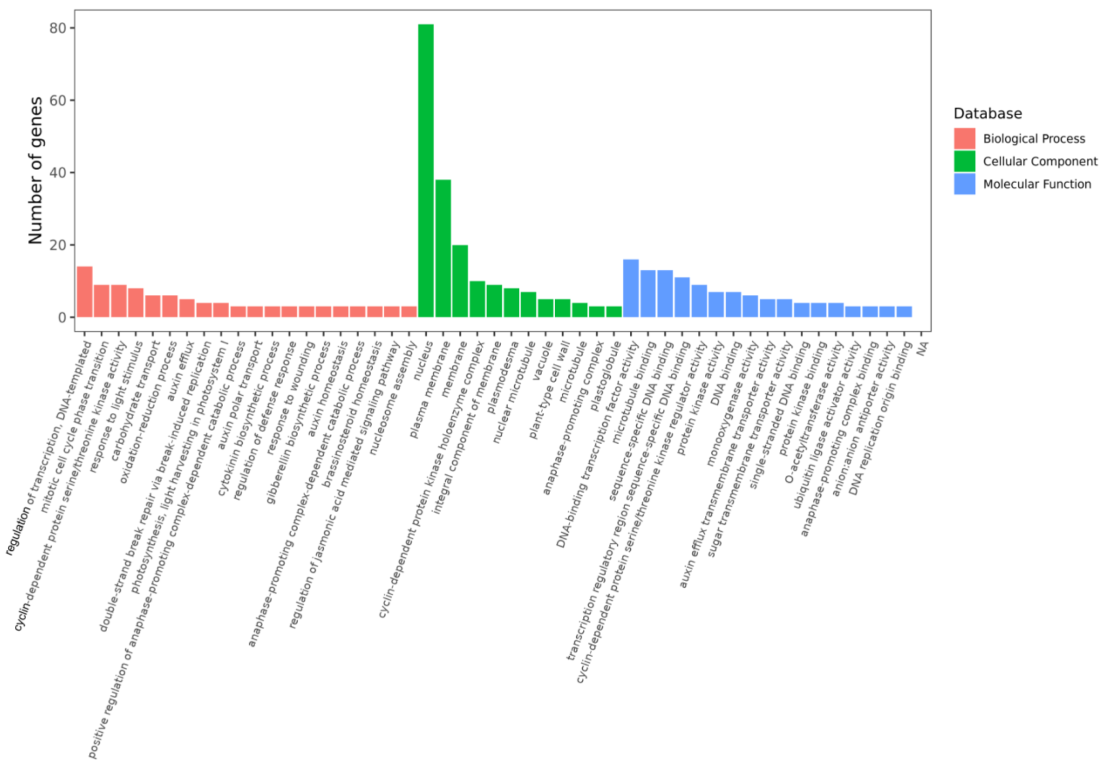

Supplementary Fig. 7. GO enrichment analysis of differentially expressed genes in the CR-*pt*^{H} and wild-type TS-9 lines.

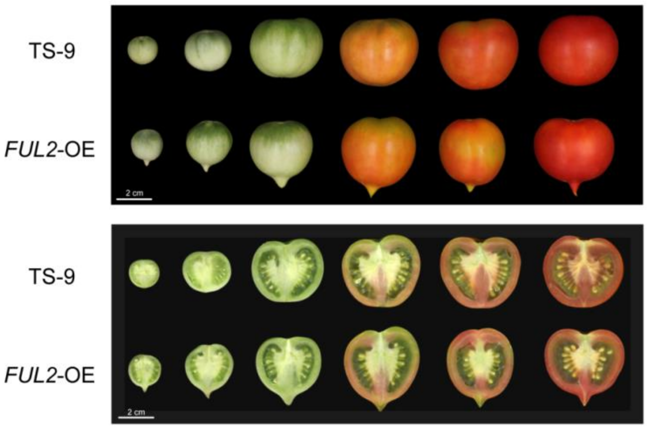

Supplementary Fig. 8. Fruit phenotypes of *FUL2* overexpression lines (*FUL2*-OE) and the wild-type control (TS-9).

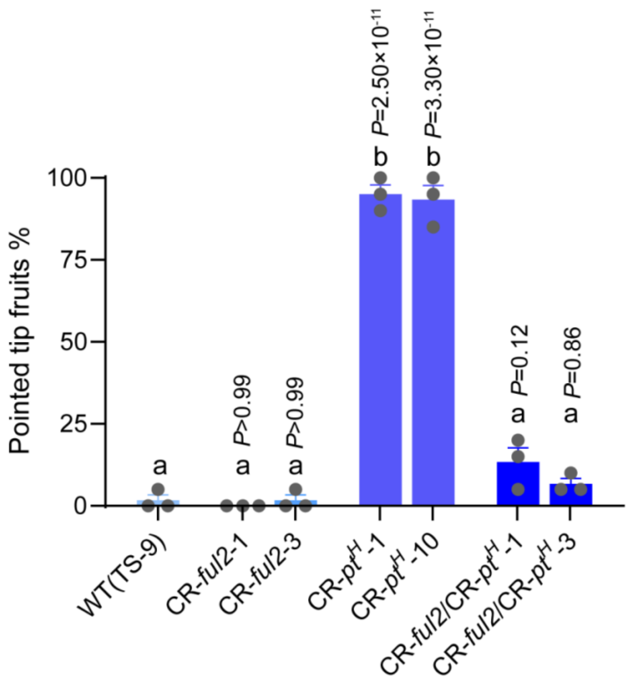

Supplementary Fig. 9. Percentage of pointed tip fruit produced by different mutants and wild-type TS-9.

20 fruits from each replicate were harvested and recorded for the morphologies (pointed tip or non-pointed tip). Error bars indicate mean ± SE. *n*=three biological replicates. Statistically significant differences were determined using a one-way ANOVA with Tukey's post-hoc test. Different letters indicate statistically significant differences ($P < 0.05$). Source data are provided as a Source Data file.

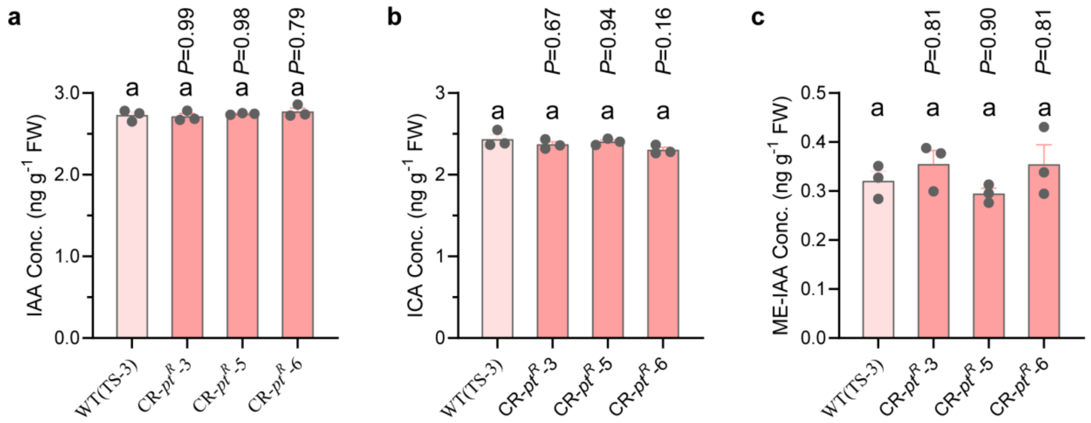

Supplementary Fig. 10. Auxin content of pointed tips in CR-*pt*^{R} and wild-type TS-3 lines.

Indole-3-acetic acid (IAA, **a**), indole-3-carboxaldehyde (ICA, **b**) and methyl indole-3-acetate (ME-IAA, **c**) content in the distal end of fruit from CR-*pt*^{R} and wild-type (TS-3, *PT*^{R} allele) were measured using liquid chromatography-tandem mass spectrometry (LC-MS/MS). The fruit was harvested at 14 DPA. Error bars indicate mean ± SE. *n*=three biological replicates. Statistically significant differences were determined using a one-way ANOVA with Tukey's post-hoc test. Different letters indicate statistically significant differences (P < 0.05). Source data are provided as a Source Data file.

Supplementary Table 1. List of primers used for genotyping individuals from the F2:3 population.

Marker type	Maker name	Enzyme	Annealing temp (°C)	Primer sequence(5'-3')	
CAPS	BK2	*Bsp119I*	55	F	TTTTATTGGTCCACGAGCCG
				R	AAAAATCTATGTCCAAACGAGCC
CAPS	BK72	*SspI*	55	F	TATTATGTTGCTGAGCAAAAGGC
				R	TTGTGGGGTTAAAGTGGAGAAGT
CAPS	CK9	*NcoI*	55	F	AGCTTGACTTGGTGATAGAGACC
				R	GGCGTTCTGTGCTGAAAACA
CAPS	CK20	*SacI*	55	F	CCGAAGAGCTTGCTCCTGTA
				R	AGGGCGGGAAAACTTGTCTT
CAPS	EK6	*PstI*	55	F	CGAGACCACGTGCTTAACCA
				R	CCAGTGCCTTTGTGTTTGCC
CAPS	EK12	*VspI*	55	F	TCACCGTTACCAATTTCACCAT
				R	GCTCTTAGTTCCTACATCTCCAAGTT
