# Supplementary material for: Variation in the fruit development gene POINTED TIP regulates protuberance of tomato fruit tip
Source: Nat Commun. 2022 Oct 8;13:5940. doi: 10.1038/s41467-022-33648-4 (PMC9547884; doi:10.1038/s41467-022-33648-4)
Supplement: Supplementary file 3 — Description of Additional Supplementary Files [file 41467_2022_33648_MOESM3_ESM.pdf]

Description of Additional Supplementary Files

Supplementary Data 1

Description: Accessions used in this study and the phenotypic data.

Supplementary Data 2

Description: Loci identified using a GWAS that regulate pointed tip fruit.

Supplementary Data 3

Description: Amino acid sequences of PT^{R} and PT^{H}.

Supplementary Data 4

Description: Amino acid sequences of the CRISPR-Cas9-engineered *pt* mutants.

Supplementary Data 5

Description: Differentially expressed genes in pointed tip of CR-pt^{H} and wild-type TS-9 lines.

Supplementary Data 6

Description: Amino acid sequences of the CRISPR-Cas9-engineered *ful2* mutants.

Supplementary Data 7

Description: List of primers used in this study.
