# Supplementary material for: Variation in the fruit development gene POINTED TIP regulates protuberance of tomato fruit tip
Source: Nat Commun. 2022 Oct 8;13:5940. doi: 10.1038/s41467-022-33648-4 (PMC9547884; doi:10.1038/s41467-022-33648-4)
Supplement: Supplementary file 11 — Reporting Summary [file 41467_2022_33648_MOESM11_ESM.pdf]

nature portfolio

Corresponding author(s): Junhong Zhang

Last updated by author(s): Sep 26, 2022

Reporting Summary

Nature Portfolio wishes to improve the reproducibility of the work that we publish. This form provides structure for consistency and transparency in reporting. For further information on Nature Portfolio policies, see our Editorial Policies and the Editorial Policy Checklist.

Statistics

For all statistical analyses, confirm that the following items are present in the figure legend, table legend, main text, or Methods section.

n/a	Confirmed	
☐	☒	The exact sample size (*n*) for each experimental group/condition, given as a discrete number and unit of measurement
☐	☒	A statement on whether measurements were taken from distinct samples or whether the same sample was measured repeatedly
☐	☒	The statistical test(s) used AND whether they are one- or two-sided<br>*Only common tests should be described solely by name; describe more complex techniques in the Methods section.*
☒	☐	A description of all covariates tested
☐	☒	A description of any assumptions or corrections, such as tests of normality and adjustment for multiple comparisons
☐	☒	A full description of the statistical parameters including central tendency (e.g. means) or other basic estimates (e.g. regression coefficient) AND variation (e.g. standard deviation) or associated estimates of uncertainty (e.g. confidence intervals)
☐	☒	For null hypothesis testing, the test statistic (e.g. *F*, *t*, *r*) with confidence intervals, effect sizes, degrees of freedom and *P* value noted<br>*Give P values as exact values whenever suitable.*
☒	☐	For Bayesian analysis, information on the choice of priors and Markov chain Monte Carlo settings
☒	☐	For hierarchical and complex designs, identification of the appropriate level for tests and full reporting of outcomes
☒	☐	Estimates of effect sizes (e.g. Cohen's *d*, Pearson's *r*), indicating how they were calculated

Our web collection on statistics for biologists contains articles on many of the points above.

Software and code

Policy information about availability of computer code

Data collection	No software was used in data collection.
Data analysis	GEMMA version 0.94.1, LocusZoom version 0.12, GraphPad Prism version 8.3.0, ClustalW2.0, and MEGA version 7.0.21

For manuscripts utilizing custom algorithms or software that are central to the research but not yet described in published literature, software must be made available to editors and reviewers. We strongly encourage code deposition in a community repository (e.g. GitHub). See the Nature Portfolio guidelines for submitting code & software for further information.

Data

Policy information about availability of data

All manuscripts must include a data availability statement. This statement should provide the following information, where applicable:

- Accession codes, unique identifiers, or web links for publicly available datasets
- A description of any restrictions on data availability
- For clinical datasets or third party data, please ensure that the statement adheres to our policy

The sequence data used in this article can be found in the Sol Genomics Database (https://solgenomics.net/) with the following accession numbers: PT (Solyc05g054030), FUL2 (Solyc03g114830), PIN4 (Solyc05g008060), PIN5 (Solyc01g068410), PIN9 (Solyc10g078370), PIN10 (Solyc04g056620), LAX3 (Solyc11g013310). The genomic sequencing datasets of BSA analysis and RNA sequencing datasets generated in this study have been deposited in Sequence Read Archive (SRA) under the accession number PRJNA788715 (https://www.ncbi.nlm.nih.gov/sra/?term=PRJNA788715) and PRJNA861842 (https://www.ncbi.nlm.nih.gov/sra/?term=PRJNA861842). Other data supporting our findings are available within this manuscript and its supplementary information files. The clones and plant materials generated during this study are available from the corresponding author upon request. Source data are provided with this paper.

Field-specific reporting

Please select the one below that is the best fit for your research. If you are not sure, read the appropriate sections before making your selection.

☒ Life sciences ☐ Behavioural & social sciences ☐ Ecological, evolutionary & environmental sciences

For a reference copy of the document with all sections, see nature.com/documents/nr-reporting-summary-flat.pdf

Life sciences study design

All studies must disclose on these points even when the disclosure is negative.

Sample size	For fruit pointed tip investigations, about 20 fruits were used based on the testable hypotheses.
Data exclusions	No data was excluded.
Replication	All experiments were successfully replicated three times.
Randomization	Fruits used for measurements were collected and allocated in random at same development stage.
Blinding	Fruit sample collection was random and sample size was reasonable. Therefore, blinding is not possible.

Reporting for specific materials, systems and methods

We require information from authors about some types of materials, experimental systems and methods used in many studies. Here, indicate whether each material, system or method listed is relevant to your study. If you are not sure if a list item applies to your research, read the appropriate section before selecting a response.

Materials & experimental systems

n/a	Involved in the study	
☒	☐	Antibodies
☒	☐	Eukaryotic cell lines
☒	☐	Palaeontology and archaeology
☒	☐	Animals and other organisms
☒	☐	Human research participants
☒	☐	Clinical data
☒	☐	Dual use research of concern

Methods

n/a	Involved in the study	
☒	☐	ChIP-seq
☒	☐	Flow cytometry
☒	☐	MRI-based neuroimaging
